# The common NOD2/CARD15 variant P268S in patients with non-infectious uveitis: a cohort study

**DOI:** 10.1186/s12969-015-0037-5

**Published:** 2015-10-06

**Authors:** E. Marrani, R. Cimaz, O M Lucherini, R. Caputo, A. Vitale, L. Cantarini, G. Simonini

**Affiliations:** Paediatric Rheumatology Unit, Anna Meyer Children Hospital, University of Firenze, Florence, Italy; Research Center of Systemic Autoimmune and Autoinflammatory Diseases, Rheumatology Unit, Policlinico Le Scotte, University of Siena, Siena, Italy; Paediatric Ophthalmology Unit, Anna Meyer Children Hospital, University of Firenze, Florence, Italy

**Keywords:** Paediatric uveitis NOD2/CARD15, Inflammatory eye disease, Intra-ocular inflammation, Chronic autoimmune uveitis, Blau syndrome

## Abstract

**Background:**

The etiology of Autoimmune chronic uveitis (ACU) is still unknown; NOD2/CARD15 gene mutations are responsible for the Blau Syndrome and can induce uveitis in animal models.

**Presentation of the hypothesis:**

Aim of our study was to assess if NOD2/CARD15 variants have a role in the etiology or in the clinical course of patients with ACU, either idiopathic or associated with other inflammatory diseases.

**Testing the hypothesis:**

We consecutively enrolled 25 patients (19 pediatric and 6 adults) affected with ACU. For each patient medical history was reviewed and clinical data were recorded. Allelic and genotypic frequencies of NOD2/CARD15 variations were calculated in patients and matched with those of 25 healthy controls. The statistical analysis was performed.

Fifteen patients showed the polymorphism P268S/SNP5 (SNP rs2066842) as heterozygous carriers while two patients were homozygous for the same polymorphism; one patient carried also the variant c647 18–16 TCT on intron 3, not previously reported in the literature. Statistical analysis for NOD2/CARD15 genotyping showed significant differences between patients and controls for allelic frequencies (*p* = 0.04, OR: 4.03, 95 %; CI = 1.2–13.5) but not for genotypic frequencies. We could not identify a significant phenotype-genotype correlation.

**Implications of the hypothesis:**

In our cohort of Italian patients, the NOD2/CARD15 common variant P268S/SNP5 could potentially be significantly associated with ACU.

## Background

The term “non-infectious uveitis” encompasses a group of heterogeneous conditions characterized by an inflammatory process affecting both the uveal tract and the adjacent structures; the intra-ocular inflammation can complicate the clinical course of various autoimmune disorders, mainly Juvenile Idiopathic Arthritis, or can arise *de novo* and be limited to the eye (idiopathic uveitis) [1]. The current classification of Autoimmune Chronic Uveitis (ACU) is based on the anatomical localization of the inflammation and classify uveitis as anterior, intermediate, posterior and panuveitis [2].

Although ACU is considered a rare disorder, clinicians should not overlook it; indeed this condition is potentially sight-threatening and represents one of the most common causes of legal blindness in the developed countries. Patients affected by uveitis often experience relapses and complications and require long term immunosuppressive therapy and a multidisciplinary approach [3].

The pathogenesis of intra-ocular inflammation is still unknown, but genetic factors may have a key role in the susceptibility to some uveitis entities; therefore several studies have investigated the genetic causes of uveitis and numerous genes, involved in both innate and adaptive immune responses, have been studied [4, 5].

The Caspase recruitment domain gene *CARD15* appears to play an important role in regulating inflammatory responses against bacteria [6]; it encodes the NOD2 receptor which is a member of the pattern recognition receptors (PRRs) family, is expressed mainly in the cytoplasm of monocytes and APCs (antigen presenting cells) and can detect intracellular pathogens.

Both members of the NOD family (NOD1 and NOD2) are composed of three domains: a C-terminal leucine-rich repeat (LRR) domain involved in ligand recognition, a central nucleotide binding oligomerization domain (NOD) with ATPase-mediated self-oligomerization capacity, and an N-terminal CARD domain.

The role of the NOD2/CARD15 gene in ACU is particularly intriguing: certain mutations (mainly R334W, R334Q and L469F) alter the NOD domain of the receptor, increase the activity of the NF-kB pathways and cause a rare autosomal dominant inflammatory disease, named “Blau Syndrome”, which is characterized by early-onset granulomatous arthritis, dermatitis and recurrent uveitis [7].

On the other hand, other mutations of this gene, affecting the leucine-rich repeat domain of the protein, have been associated with an increase susceptibility to Crohn's Disease, a granulomatous inflammatory disease affecting the gastrointestinal tract and complicated by uveitis in the 5–10 % of all patients [8].

## Presentation of the hypothesis

The role of NOD2/CARD15 gene as a key regulator of innate immunity in the eye has been elucidated by studies performed on murine models,which are knockout for NOD2/CARD15, which showed an increased susceptibility to uveitis [9, 10]. However the first reports conducted on patients affected by idiopathic uveitis has failed to demonstrate a role for this gene in this disease; two studies conducted on a large cohort of spanish patients with idiopathic uveitis suggested that NOD2/CARD15 variants do not seem to predispose to thee disease [11, 12]. In order to investigate the possible role of this gene in ACU, we have studied NOD2/CARD15 variants and the genotype-phenotype correlation in a cohort of patients with idiopathic uveitis and uveitis associated with other inflammatory diseases.

## Testing the hypothesis

### Patients

Twenty-five unrelated Italian patients with an established diagnosis of ACU have been included in the present study: the population was composed of 19 pediatric and 6 adults. Patients were consecutively enrolled in 2012–2013 at paediatric hospital “Anna Meyer” (Florence, Italy) and at Hospital “Santa Maria alle Scotte” (Siena, Italy). Inclusion criteria were the presence of ACU and willingness to participate in the study. Written consent was obtained by all adult patients or guardians. Data regarding age of onset, type and localization of uveitis, associated diseases, and complications were collected and recorded with a customized database. The analysis of the exon 4 of the NOD2/CARD15 gene was also performed on the DNA of 25 tuscanian controls.

Controls have been chosen between patients referred to our centers in the 2010–2014 period with an history of mechanical or functional joint pain and submitted to DNA collection; these patients were age and sex-matched and were use as historical control population. We selected patients and controls of tuscanian ancestry, according to family history reported, in order to guarante a common genetic background.

### DNA extraction and genotyping

Blood samples were collected in EDTA-containing tubes during routine venipuncture. DNA was extracted using the High Pure PCR Template Preparation kit (Roche Applied Science, France), according to manufacturer’s instructions. Genomic DNA was eluted in sterile water, and stored at −20 °C; the concentration of the eluted genomic DNA was determined by spectrophotometry with the Nanodrop® ND-1000 (NanoDrop Technologies INC, Wilmington, DE, USA). PCR reaction was conducted using primers designed with the Primer3 software. Samples were amplified in a Eppendorf Mastercycler thermal cycler and then the PCR products were purified using the WizardSVGel and PCR Clean-up System (Promega). The purification was applied to both PCR product and extracts from agarose gel. The sequencing reaction was performed with ABI 3730 DNA Analyzer (Applied Biosystems Life Sciences Corporation, Carlsbad, CA, U.S.A.) and the electropherogram analysis was performed through the software Sequencher v.4.2.2 (GeneCodes, USA).

### Statistical analysis

Allelic and genotypic frequencies (CC, CT, TT) in cases and controls were calculated; then differences between groups in terms of SNP variants frequencies were determined with chi-square test and Fisher exact test with Monte Carlo simulations, when appropriate. Since the number of subjects carrying a TT genotype was very low, the analysis was performed also comparing frequency data in two groups: those carrying at least one CARD 15 *T allele and those carrying the CC genotype. The Spearman rank correlation test was used to determine correlation coefficients for different variables [gender, age at diagnosis, uveitis duration, types of uveitis (granulomatous, and not-granulomatous, anterior, posterior, pars-planitis, mono/bilateral), ANA positivity, HLA-B27 presence, auto-immune associated diseases, eye complications]. The odds ratio (OR) and 95 % confidence interval (95 % CI) were calculated. Non-parametric tests were used, where necessary, due to the small size of our groups and to the skewness of our data. Levels of *p* < 0.05 were considered statistically significant. All analyses were performed on SPSS package for Windows, version 13.0 (SPSS, Inc., Chicago, IL, USA).

### Prevalence of NOD2/CARD15 variants

The P268S/SNP5 (SNP rs2066842) involves a C to T change resulting in a P to S amino acid change; these common variation of the NOD2/CARD15 gene have been identified in 17 of our cases: 15 patients showed the polymorphism P268S/SNP5 (SNP rs2066842) as heterozygous carriers while 2 patients were homozygous for the same polymorphism. The polymorphism distribution in AUC resulted as follows: 6/25 CC (24 %), 17/25 CT (68 %), 2/25 TT (8 %) [Table 1]. One patient carried two different mutations: the polymorphism P268S/SNP5 in heterozygous and a variant on intron 3 (c647 18–16 TCT). We regarded the variation c647 as a non-functional one and so we include this patient in the same group as the heterozygous carriers of the P268S/SNP5 polymorphism. The incidence of P268S/SNP5 in our population was compared with the incidence reported for 25 healthy historical controls, age and sex-matched. According to genotype analysis, 14/25 controls (56 %) did not present this polymorphism, 10/25 (40 %) were heterozygous carrier and 1/25 (4 %) were homozygous. No evidence of departure from Hardy-Weinberg equilibrium in controls was seen (*p* = 0.65).

**Table 1:** Comparison of CARD 15* genotype frequencies (%) in Autoimmune Chronic Uveitis versus controls

Genotype	Cases (*n* = 25)
CARD 15*TT	2 (8 %)
CARD 15*TC	17 (68 %)
CARD 15* CC	6 (24 %)

Values are the number (%) of the subjects tested

CARD 15* genotype comparison among controls and patients. (*p* = 0.06)

Statistical analysis for CARD 15 genotyping showed significant differences between patients and controls for allelic frequencies but not for genotypic frequencies. T allele was present in 19/25 (76 %) AUC subjects compared to 11/25 (44 %) controls, and C allele in 23/25 (33.3 %) (*p* = 0.04, OR: 4.03, 95 % CI = 1.2–13.5) [Table 2].

**Table 2:** A Comparison of CARD 15* C/T allele frequencies (%) in Autoimmune Chronic Uveitis versus controls

Genotype	Cases (*n* = 25)
CARD 15* T	19 (76 %)
CARD 15* C	6 (24 %)

Values are the number of subjects tested

CARD 15* T allele resulted more frequent in Autoimmune Chronic Uveitis than in controls (*p = 0.04, OR: 4.03, 95 % CI = 1.2-13.5)

The functional role of the P268S/SNP5 (SNP rs2066842) variant was predicted using SIFT (version 5.0.2) and PolyPhen (PolyPhen-2 version 2.2.2, release 405).

### Genotype-phenotype correlation

Table 3 show the main characteristics of our cohort of enrolled patients. We also tried to assess if the T allele presence could impact the clinical course of the disease in patients. We failed to show any association between one genotype and the considered clinical variables. In particular, outcome of ACU was not modified by NOD2/CARD15 polymorphisms, since the incidence of macular edema, synechiae, glaucoma, visual loss and cataract was not increased in the group of patients heterozygous or homozygous for P268S/SNP5 [Table 4].

**Table 3:** Demographical and medical characteristics of the studied population

Characteristics	Pediatric Population (*n* = 18)	Adult population (*n* = 7)
Age		
Mean ± SD	11,8 ± 4,5 years	50 ± 22 years
Median (range)	11 (4 to 17 years)	53 (21 to 76 years)
Female gender, n (%)	12 (66 %)	5 (71 %)
Systemic disease association		
None	8	2
Arthritis (JIA, Spondyloarthropathies, other forms)	10	2
Other	/	3
Family history of autoimmune or inflammatory disease, n (%)	5 (27 %)	1 (14 %)
Uveitis		
Age of onset		
Mean ± SD	6,6 years ± 3	45 years ± 22
Median (range)	6,6 years (4 to 15 years)	50 (11 years to 74 years)
Duration		
Mean ± SD	5,2 ± 4,4 years	4,9 ± 3,2 years
Median (range)	3,8 years	4,1 years

**Table 4:** Phenotype-genotype correlation

	No mutation	He	Ho	*p-value*
		P268/SNP5	P268/SNP5	
Comorbility, % (n)	5	9	50 % (1)	n.s.
Positive family history, % (n)	1	4	50 % (1)	n.s.
Granulomatous uveitis, % (n)	0	5	0	n.s.
Panuveitis, % (n)	3	9	0	n.s.
Bilateral uveitis, % (n)	6	8	50 % (1)	n.s.
ANA positive, % (n)	3	7	50 % (1)	n.s.
HLA B27 positive, % (n)	0	2	0	n.s.
Macular edema, % (n)	3	7	0	n.s.
Synechiae, % (n)	2	4	1	n.s.
Glaucoma, % (n)	2	2	0	n.s.
Visual loss, % (n)	2	3	0	n.s.
Cataract, % (n)	2	1	0	n.s.
He = heterozygous; Ho = homozygous				n.s.

## Implications of the hypothesis

ACU appears as an heterogeneous group of disorders resulting from a disregulation of both innate and adaptative immune responses; the genetic bases of these conditions appear to be extremely complex. The interesting hypothesis that NOD2/CARD15 mutations can trigger ocular inflammation has been suggested by several studies, mostly carried out in animal models [9, 10]: in mice NOD2 exerts a regulatory role on the inflammatory process and may participate in the development of uveitis in response to bacterial peptidoglycans. It has been shown that in humans this association is more complex and not fully understood; researchers' interest on this gene has been evoked by the discovery that loss-of-function mutations and gain-of-function mutations can be both identified in two different diseases, Crohn's disease and Blau syndrome, showing non-caseating epithelioid granulomas as prominent histopathological features.

In Blau syndrome mutations (mainly R334W, R334Q and L469F) alter the NOD domain of the receptor and exert a direct pathogenetic role, and uveitis is part of the diagnostic triad; it is a distinctive granulomatous, both anterior and posterior, and its prognosis in term of visual loss is grim because it can evolve into a severe destructive panuveitis and be responsible for permanent blindness. In Crohn's disease mutations affecting the leucine-rich repeat domain of the receptor confer to carriers an increased susceptibility to the disease and chronic anterior uveitis is an extra-intestinal feature of this condition.

The variant P268S/SNP5 (SNP rs2066842) involves a C to T change resulting in a P to S amino acid change; according to SIFT (version 5.0.2) and PolyPhen (PolyPhen-2 version 2.2.2, release 405) software software; predictions this variant appeared to be tolerated without deleterious impact on protein function.

In our ACU population the variant P268S/SNP5 (SNP rs2066842) was found in 17 over 25 patients; the prevalence of our variant was compared with the incidence reported for 25 healthy historical controls, age and sex-matched.

In contrast to previous reports [11, 12], our study, showed a statistical association between the variant P268S/SNP5 (SNP rs2066842) and ACU, both idiopathic and associated with other rheumatic disease. In our cohort the incidence of variations was not significantly different between our patients with idiopathic chronic uveitis and those with chronic uveitis associated to auto-immune diseases.

However, our analysis failed to find any significant association between CARD 15 mutation and type, clinical course and outcome of our cohort of ACU.

Other studies trying to link NOD2 mutations and non-infectious chronic uveitis failed to show any association, with the remarkable exception of the Blau related ones. In particular, Rodriguez-Perez and colleagues [10, 11], in their cohort of more than 100 patients with idiopathic chronic uveitis, reported allele frequencies as high as 50 % for the variant P268S, 7 % for the mutation R702W and 2 % for the mutation G908R while none of the mutations responsible for Blau syndrome (R334W, R334Q and L469F) was found in any of the individuals tested. When the frequency of the different mutations in the group of patients was compared to that of 104 healthy subjects, no statistical differences were found and the authors concluded that none of the CARD15 mutations linked to Crohn's Disease was involved in the pathogenesis of idiopathic uveitis.

Our study enrolled only a limited cohort of patients with ACU and we decided to adopt a case-control study design; thus the statistic power of our study is to be considered limited. However we can't rule out a permissive effect of the NOD2 common variant in the development of uveitis.

This study was desgned as a pilot study; a study involving more patients affected with ACU is currently ongoing in order to clarify if a different genetic background between cases as well a sample bias can explain this difference in P268S/SNP5 (SNP rs2066842) frequency between the cases and controls.

However our healty controls showed an incidence comparable with the data reported in the literature and in the genomic databases for the Tuscanian population (ref.http://www.ensembl.org/Homo_sapiens/Variation/Population?db=core;r=16:50744124–50745124;v=rs2066842;vdb=variation;vf=1636572#).

The variants c647 has not previously reported in the literature and its role on gene's function is unknown. Further functional analyses are needed in order to disclose its clinical significance.

To our knowledge, this is the first report demonstrating a relationship between a NOD2 variants and eye inflammation; nevertheless our analysis could not identify any phenotype-genotype correlation. Moreover we could not assess the influence of the genotype on the response to pharmacological treatment because of the wide range of therapies used in these patients. Further studies should include a larger number of patients in order to identify the genetic risks factors of non-infectious uveitis.
